# Human Granulocytic Anaplasmosis—A Systematic Review of Published Cases

**DOI:** 10.3390/microorganisms10071433

**Published:** 2022-07-15

**Authors:** Igor Dumic, Dorde Jevtic, Mladjen Veselinovic, Charles W. Nordstrom, Milan Jovanovic, Vanajakshi Mogulla, Elmira Mofid Veselinovic, Ann Hudson, Gordana Simeunovic, Emilia Petcu, Poornima Ramanan

**Affiliations:** 1 Mayo Clinic Alix School of Medicine, Rochester, MN 55905, USA; nordstrom.cw@mayo.edu (C.W.N.); mogulla.vanajakshi@mayo.edu (V.M.); hudson.ann@mayo.edu (A.H.); petcu.emilia@mayo.edu (E.P.); 2 Department of Hospital Medicine, Mayo Clinic Health System, Eau Claire, WI 54703, USA; 3 Icahn School of Medicine at Mount Sinai, New York, NY 10029, USA; djordje965@gmail.com; 4 Internal Medicine Department, Elmhurst Hospital Center, New York, NY 11373, USA; 5 Infectious Disease Department, Baptist Health Medical Center, North Little Rock, AR 72117, USA; proximus.7@gmail.com; 6 School of Medicine, University of Belgrade, 11000 Belgrade, Serbia; jovanovic.milan@gmx.com; 7 Baptist Health Medical Center, North Little Rock, AR 72117, USA; elmira.mofid@gmail.com; 8 Infectious Disease Department, Spectrum Health/Michigan State University, Grand Rapids, MI 49503, USA; gordana.simeunovic@spectrumhealth.org; 9 Infectious Disease Department, University of Colorado, Denver, CO 80204, USA; poornima.ramanan@cuanschutz.edu

**Keywords:** anaplasmosis, human granulocytic anaplasmosis, *Anaplasma phagocytophilum*, ticks, tick borne disease, coinfection, *Borrelia burgdorferi*

## Abstract

*Anaplasma phagocytophilum* is an emerging, Gram-negative, obligate intracellular pathogen that is transmitted by a tick vector. Human infection ranges from asymptomatic to severe disease that can present with pancytopenia, multiorgan failure, and death. The aim of this systematic review is to analyze case reports and case series reported over the last two decades in peer-reviewed journals indexed in the Medline/PubMed database according to the PRISMA guidelines. We found 110 unique patients from 88 case reports and series. The most common mode of transmission was tick bite (60.9%), followed by blood transfusion (8.2%). Infection was acquired by blood transfusion in nearly half (42%) of the immunocompromised patients. Most patients reported fever (90%), followed by constitutional (59%) and gastrointestinal symptoms (56%). Rash was present in 17% of patients, much higher than in previous studies. Thrombocytopenia was the most common laboratory abnormality (76%) followed by elevated aspartate aminotransferase (AST) (46%). The diagnosis was most commonly established using whole-blood polymerase chain reaction (PCR) in 76% of patients. Coinfection rate was 9.1% and *Borrelia burgdorferi* was most commonly isolated in seven patients (6.4%). Doxycycline was used to treat 70% of patients but was only used as an empiric treatment in one-third of patients (33.6%). The overall mortality rate was 5.7%, and one patient died from trauma unrelated to HGA. The mortality rates among immunocompetent and immunocompromised patients were 4.2% (*n* = 4/95) and 18.2% (*n* = 2/11), respectively. Four of the six patients who died (66.6%) received appropriate antibiotic therapy. Among these, doxycycline was delayed by more than 48 h in two patients.

## 1. Introduction

In 1990, a man died in Wisconsin, the U.S. following a nonspecific illness characterized by fever, headache, myalgias, arthralgias, acute renal failure, thrombocytopenia, and coagulopathy. While his blood cultures remained negative, a peripheral blood smear demonstrated granulocyte inclusions [1]. Over the following years, additional patients with similar symptoms and laboratory findings were identified [2]. In some of these patients, the illness manifested after a variable timeframe following a reported tick bite. In subsequent years, Dumler and his coauthors, through genetic and serologic testing, identified that the responsible organism was very similar to *Ehrlichia chaffeensis*, the cause of human monocytic ehrlichiosis (HME). Hence, this disease was first named human granulocytic ehrlichiosis (HGE) [1,2,3]. In 2001, this pathogen was renamed *Anaplasma phagocytophilum* (AP), the causative agent of human granulocytic anaplasmosis (HGA) [4].

AP is a Gram-negative, obligate intracellular rickettsial pathogen, and is transmitted by the *Ixodes* tick within the United States. Differing subspecies of the *Ixodes* tick vector have been identified in various regions: *Ixodes scapularis* in the United States Northwest and Upper Midwest; *Ixodes pacificus* in the United States Pacific Northwest; and *Ixodes ricinus* in Europe. The most common reservoirs for AP are white-tailed deer and white-footed mouse. HGA is usually a mild illness and is frequently subclinical. In some patients, however, the disease may be serious with severe cytopenias, elevated liver function tests (LFT), coagulopathy, renal failure, and even death [5,6,7,8,9,10]. Within the U.S., HGA is most prevalent in the Northeast and Upper Midwest—particularly in Wisconsin’s northwest region [1,2,3,4,5,6,7,8,9,10]. HGA is an emerging infection and an increase in its incidence is expected in the coming years [11].

The aim of this systematic review is to describe the clinical features, diagnosis, treatment, and outcomes of patients with HGA by extensively analyzing case reports and case series published over the last 20 years according to preferred reporting items systematic review and meta-analysis (PRISMA) guidelines.

## 2. Materials and Methods

**Figure 1:**
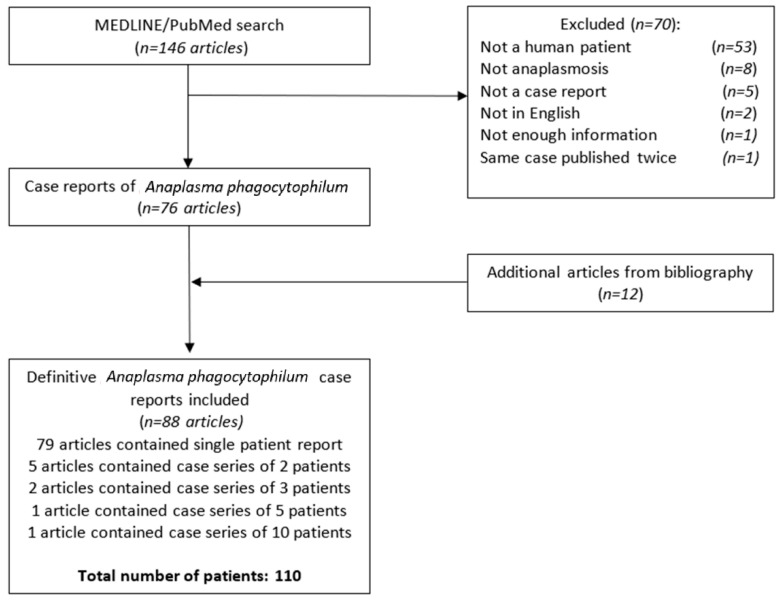
PRISMA flow chart.

We used PRISMA guidelines to select articles eligible for inclusion. The keywords used were ‘anaplasmosis’ and ‘human granulocytic anaplasmosis’. Two authors (D.J. and M.V.) independently searched the MEDLINE/PubMed database using the above-described keywords from January 2002 to September 2021. The discrepancies were resolved with the assistance of a senior author (ID). We recognize that before 2001, this disease was named HGE, but this term was not used as we limited our search to the past two decades. The PRISMA flow chart is illustrated in Figure 1. References of included articles were reviewed to include additional articles that might have been missed during the initial database search. Ultimately, our systematic review included 88 articles and 110 patients in total.

Patients were considered immunocompromised if they had any of the following: active malignancy treated with chemotherapy and/or radiation, transplant recipients on antirejection therapy, patients with acquired immunodeficiency syndrome (AIDS), treatment with steroids, asplenia, untreated or poorly controlled diabetes mellitus (DM), or chronic kidney disease (CKD) requiring renal replacement therapy.

Constitutional symptoms were defined as malaise, weakness, or fatigue. Duration of illness was defined as the number of days from symptom onset until symptom resolution. For laboratory data, the highest values were reported for blood urea nitrogen (BUN), creatinine (Cr), bilirubin, aspartate aminotransferase (AST), alanine aminotransferase (ALT), alkaline phosphatase (ALP), lactate dehydrogenase (LDH), C-reactive protein (CRP), erythrocyte sedimentation rate (ESR), and ferritin. The lowest laboratory values were reported for sodium, hemoglobin (Hb), platelets, and white blood cells (WBC).

## 3. Results

### 3.1. Demographic Characteristics

**Figure 2:**
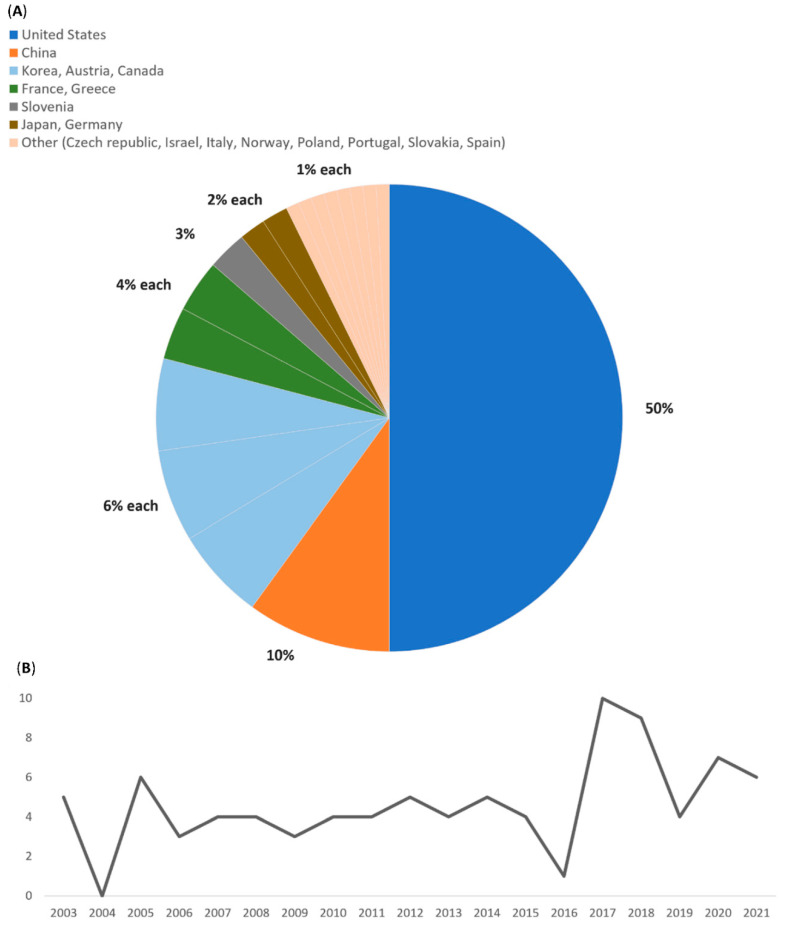
(**A**) List of countries where the case reports originated. (**B**) Number of article years.

Of 110 patients, 58 (52.7%) were male, and 9 (8.2%) did not have a gender reported [6,12,13,14,15,16,17,18,19,20,21,22,23,24,25,26,27,28,29,30,31,32,33,34,35,36,37,38,39,40,41,42,43,44,45,46,47,48,49,50,51,52,53,54,55,56,57,58,59,60,61,62,63,64,65,66,67,68,69,70,71,72,73,74,75,76,77,78,79,80,81,82,83,84,85,86,87,88,89,90,91,92,93,94,95,96,97,98]. The mean age was 54.6 years (range 1–85), and 51 patients (46.4%) had at least one comorbidity. Fifteen patients (13.6%) were immunocompromised due to steroid use (*n* = 5, 4.5%), DM (*n* = 5, 4.5%), CKD (*n* = 4, 3.6%), treatment with cytotoxic medication (*n* = 3, 2.7%), active hematologic malignancy (*n* = 3, 2.7%), or asplenia (*n* = 1, 0.9%). Six patients (5.5%) had more than one cause of immunosuppression. The complete list of comorbidities is presented in Table 1. The countries with the highest incidence of cases are the U.S. (*n* = 55, 50%), China (*n* = 11, 10%), Korea (*n* = 7, 6%), Austria (*n* = 7, 6%), and Canada (*n* = 7, 6%). A complete list of countries is presented in Figure 2A. We did not find that the number of reported cases has increased over the years; however, there was a relatively higher number of cases reported in the literature in the period 2016–2018 (Figure 2B).

### 3.2. Exposure and Clinical Presentation

The most frequent mode of transmission was tick bite, which was either confirmed or highly suspected in 67 cases (60.9%). The majority of these patients participated in outdoor activities, such as hunting, gardening, or hiking through forests. Nine patients (8.2%) were infected through blood transfusion, and nine patients (8.2%) reported exposure to blood or bloody respiratory secretions as a possible source of infection. In the remaining 25 cases (22.7%), the mode of transmission was not reported.

Fever was the most frequently reported symptom in 99 patients (90%). Constitutional symptoms were present in 65 (59.1%), myalgias in 46 (41.8%), headache in 41 (37.3%), and chills in 35 patients (31.8%). Gastrointestinal (GI) symptoms were highly prevalent as well, with 61 patients (55.5%) reporting at least one of the following: diarrhea, nausea, vomiting, anorexia, or abdominal pain. The rash was present in 17% of patients. The complete list of common symptoms is reported in Table 2. Duration of illness was reported in 53 cases, with the mean duration being 20.8 days (range 5–105). Duration of hospitalization was reported in 46 cases, with the mean number of days in hospital being 14.6 days (range 2–50).

### 3.3. Investigations (Laboratory, Pathogen Identification)

The following laboratory parameters were analyzed: Hb, platelets, WBC, BUN, Cr, bilirubin, LFTs, sodium, LDH, CRP, ESR, and ferritin. Anemia was present in 33 (30%), leukopenia in 51 (46.4%), and thrombocytopenia was the most prominent cytopenia in 79 patients (71.8%). The most common LFT abnormality was a high level of AST in 63 patients (57.3%). CRP was elevated in 37 patients (33.6%). Detailed laboratory data and observed abnormalities are presented in Table 3.

Diagnosis of HGA was confirmed using whole-blood polymerase chain reaction (PCR) in 79 patients (71.8%), serology in 73 patients (66.4%), peripheral blood smear in 48 patients (43.6%), and isolation of AP in culture in 10 patients (9.1%). Five cases (4.5%) did not report the modality used for pathogen identification.

Coinfection with two or more pathogens was identified in 10 patients (9.1%). The most common coinfection was with *Borrelia* spp. in seven patients (6.4%). Two patients had tick-borne infections caused by three pathogens: *AP, Borrelia Burgdoferi* (BB), and *Babesia microti* (BM).

### 3.4. Therapy

Doxycycline is the mainstay of treatment and was used in 77 patients (70%). The duration of doxycycline therapy was assessed in 52 cases, and the mean number of days was 12.9 (range: 6–30). Empiric antibiotics were commonly utilized before disease confirmation and the most common ones were: ceftriaxone (*n* = 23, 20.9%), vancomycin (*n* = 19, 17.3%), and azithromycin (*n* = 11, 10%). Doxycycline was reported as a part of the empiric therapy before diagnosis confirmation only in 37 patients (33.6%). Rifampin was commonly used instead of doxycycline in pediatric and pregnant patients (*n* = 9, 8.2%). Less commonly employed therapies included: intravenous immunoglobulins (IVIG) (*n* = 3, 2.7%), platelet transfusion (*n* = 2, 1.8%), and erythrocytapheresis (*n* = 1, 0.9%).

### 3.5. Outcome

Out of the 110 patients in our review, the outcome was described in 106 patients (96.4%). A total of 6 out of 106 patients passed away (5.7%), and 1 patient died from trauma unrelated to HGA. Five patients who died were male (83.3%), and four (66.6%) had at least one comorbidity. Four of six patients who died (66.6%) received appropriate antibiotic therapy, while in the other two the therapy was inappropriate. In the two patients who died and received doxycycline (50%), treatment was delayed for more than 48 h. While the overall mortality rate was 5.7%, it was 4.2% (4/95) and 18.2% (2/11) among immunocompetent and immunocompromised patients, respectively.

## 4. Discussion

### 4.1. Epidemiology and Pathophysiology

Epidemiological data suggest that cases of HGA in the U.S. have increased significantly over the years, from 348 cases in 2000 to 5655 cases in 2019, with the mortality rate remaining relatively stable at 0.6–1% [8,11]. One of the possible reasons for this observed increase in HGA cases is climate change contributing to an increase in arthropod vectors and vertebrate host reservoirs [8,99]. Similar to other studies (5–8100), we also found that most reported cases of HGA were in men older than 40.

AP has been reported in the U.S., Asia [67,68,75,77,78,97], and Europe [12,23,24,32,46,57,65,69]. Slovenia has the highest seroprevalence (17%), followed by northwest Wisconsin (15%) and Sweden (12%) [100]. Within the U.S., the states with the most reported cases were Connecticut, New York, Rhode Island, and Wisconsin [8]. HGA has been recently recognized as an etiology of fever in returning travelers with one study demonstrating an incidence rate of 19.9 cases/1000 person-weeks of travel [101]. Most of these patients had returned from Asia (62.5%) and all reported activities in rural areas with increased risk for tick exposure [101]. Interestingly, as providers did not suspect HGA, only 25% of patients were appropriately treated with empiric doxycycline. These findings are similar to our review of cases in which doxycycline was reported as a part of an empiric antimicrobial regimen in only one-third of patients.

While tick bite remains the primary mode of transmission, up to 25% of patients do not report a tick bite [5]. AP can also be transmitted vertically (transplacental), by direct contact with infected blood (human or animal), and has been documented to be a nosocomial pathogen [5,97,102,103]. We found that a tick bite was confirmed or highly suspected in 67 patients (60.9%) which correlates with previous reports showing that not all patients are able to recall tick bites.

Nine patients (8.2%) were infected through blood transfusion. Blood products are not routinely screened for the presence of AP and techniques such as leukoreduction were previously considered effective by eliminating leukocytes infected with causative organisms. However, one study reported that despite leukoreduction, AP was transmitted by transfusion in 83% of blood components [104]. It is interesting to note that 42.9% of immunocompromised patients were infected through blood transfusion. It has been postulated that transfusion-related HGA might be indolent and asymptomatic in immunocompetent individuals, and it is the state of immunosuppression that leads to a clinically symptomatic illness in this patient population [15,42,51]. One case series reported exposure to a single patient’s blood and bloody respiratory secretions as the cause of infection in nine individuals (8.2%) [97]. All of the infected individuals were hospital staff or family, taking care of the index patient in the final 12 h before her death.

AP primarily infects neutrophils (PMN) due to its ability to survive and multiply within the PMN’s cytoplasmic vacuoles by delaying apoptosis and blocking programmed cell death. Additionally, AP has a specific ability to inhibit the fusion of lysosomes and cytoplasmic vacuoles, and by doing so avoids the toxic effects of neutrophils. Unlike many other Gram-negative bacteria, AP lacks lipopolysaccharide (LPS) and peptidoglycan in its cell wall which aids in its ability to evade innate host defenses. Following infection, AP stimulates the production of proinflammatory cytokines: IL-6, IL-8, IL-10, and TNF-alfa, which lead to recruitment of neutrophils, degranulation, and consequently tissue injury [5,6,7,8,56].

### 4.2. Clinical Presentation

The severity of HGA is variable; while some patients remain asymptomatic, others develop a nonspecific febrile illness, and only a minority develop severe disease. About one-third of patients with AP are hospitalized (36%) with 7% of patients requiring intensive care. The case fatality rate is around 0.6% [105]. In previous studies, the most common symptoms were fever, malaise, headache, and myalgias, and these findings are consistent with our results [3]. Unlike other studies [5], our review found gastrointestinal (GI) symptoms to be common, with 55.5% of patients reporting diarrhea, nausea, vomiting, anorexia, or abdominal pain. The most common GI symptom was diarrhea in 20 patients (18.2%). Some of the observed differences might be related to different strains of AP. For example, European strains of AP are more heterogenous compared to the American strains. Furthermore, these differences might be responsible for observed variations in mortality and severity of clinical presentations [106]. The authors of this study also commented that European cases might be relatively milder in comparison to cases from the United States; however, they too documented a relatively high hospitalization rate of almost 63% [106].

Rash is a less common presentation of HGA and is seen less frequently compared to other tick-borne diseases transmitted by *Ixodes scapularis* (i.e., Lyme disease (LD) and Ehrlichiosis). The incidence of rash in HGA was 6–10% in earlier studies [5,6,7,8], while it was 17% in our group of patients. Infection by *Anaplasma capra*, first detected in China in 2015, is more likely to cause a rash/eschar (36% of patients) [100,107] compared to AP. In a study from Poland, in 10 patients with erythema migrans (EM), BB was not isolated from the skin lesions or blood, but AP DNA was isolated from the skin biopsy. This suggests that EM might occur due to a coinfection or be caused solely by AP rather than BB [108].

Uncommon presentations included: myocarditis, seizures, short-term memory impairment, orchitis, glomerulonephritis, myositis with severe rhabdomyolysis, peripheral neuropathy at the site of a tick bite, cerebral infarct, Sweet syndrome, and hemophagocytic lymphohistiocytosis (HLH) [35,38,39,63,81,86,90,91].

It should be noted that in animals, infection might persist despite the relative lack of clinical signs and disease might recur after spontaneous recovery [109,110,111]. While spontaneous recurrence has not been observed in the reports we analyzed, spontaneous recovery, even in the absence of antimicrobial therapy, has been documented, particularly in European cases [106].

### 4.3. Coinfection

In the United States, Ixodes ticks serve as vectors for multiple human pathogens including: *Borrelia burgdorferi*, *A. phagocytophilum*, *Ehrlichia chaffeensis*, *Borrelia mayonii*, *Borrelia miyamotoi*, *Ehrlichia muris eauclairensis*, Powassan virus (POWV), and the parasite *Babesia microti* [112]. The minimum attachment required for Ixodes scapularis tick to transmit AP, *B. burgdoferi*, *B. mayonii*, and *B. miyamotoi* is 24 h [113]. In comparison, POWV can be transmitted as little as 15 min after tick attachment [113].

These ticks are frequently coinfected with two or more pathogens, and the prevalence of coinfection depends on geography. For example, in the U.S., coinfections are more common in the Northeast than in the Midwest [114]. Coinfection of BB with either AP or *Babesia microti* was the most common, seen in approximately 3% of examined ticks [114]. In Europe, coinfection of *I. ricinus* ticks varies by location. For example, studies from Ukraine showed that 12.5% of ticks were coinfected with AP and BB, while in Norway, it was significantly less at 3.3% [115,116].

The severity of illness in patients with coinfection, compared to AP monoinfection, remains unclear due to conflicting results from different studies. Horwitz et al. showed that the frequency and severity depend on how HGA is defined [117]. Coinfection rates were 2.3% based on culture results, but as high as 10% based on culture or positive antibody titer above 1:640 [117]. They found that the total number of symptoms did not vary between the monoinfection group and those coinfected with BB. However, the coinfected group had a lower frequency of headache, and lower average and maximum temperature. A study from Poland found that coinfected patients also had a lower average temperature and less severe cytopenias [108]. They demonstrated that 10% of patients with erythema migrans were coinfected with AP [108]. One study [118] showed that patients coinfected with LD and HGA were more symptomatic, but another [118] failed to demonstrate this observation. A study from Barcelona that examined the etiology of fever in returning travelers found that up to 35% of patients with HGA were coinfected with other vector-borne pathogens such as Chikungunya, *Coxiella burnettii*, and Dengue virus [101].

In our review, the rate of coinfection was 9.1%. Two patients had triple coinfections with B. burgdoferi and B. microti [35,85]. Both of these patients recovered with appropriate therapy; however, a year after, one patient reported symptoms of peripheral neuropathy at the site of a tick bite [35].

### 4.4. Diagnosis and Differential Diagnosis

Whole-blood PCR (Msp-2 gene amplification) is the most sensitive method for the diagnosis of HGA. Although blood culture remains the gold standard, it is only available in specialized labs and has lower sensitivity [5,6,7,8]. Microscopic examination of Giemsa-stained peripheral blood smear may reveal morulae (Latin for mulberry) within PMNs. Finally, serology is used for diagnosis by demonstrating *AP*-specific IgM and/or IgG antibodies by immunofluorescence. Since antibodies are not universally present during the acute phase of infection, diagnosis is confirmed by demonstrating a 4-fold increase in IgG levels, at least 4 weeks apart [5,6,7,8]. Early appropriate antimicrobial therapy or immunosuppressed state may result in false-negative serologic testing.

The differential diagnosis for HGA is very broad. It encompasses diseases transmitted by the same vector such as Lyme disease, Ehrlichiosis, Babesiosis, and POWV; however, other zoonoses and non-vector-borne diseases should also be considered.

In a febrile patient with headache, meningitis and encephalitis should be ruled out. Neuroborreliosis should be excluded, particularly as meningitis is common in early disseminated Lyme disease. POWV encephalitis is a consideration in patients who present with fever, headache, and altered mental status. Ehrlichiosis is more likely to present with neurological symptoms and rash than HGA [8]. Furthermore, patients with HME tend to be more acutely ill than those with HGA, and usually have more severe cytopenias (particularly thrombocytopenia) [8]. Hemolytic anemia is a hallmark of *Babesia microti* infection, while it is not expected to be present in HGA.

Non-tick-borne infections remain in the differential diagnosis of a febrile patient with headache, myalgias, and malaise. If a rash is present, viral exanthema (human herpesvirus 6, Epstein–Barr virus, enterovirus, adenovirus, and parvovirus B 19) and bacterial infections (endocarditis, *N. meningitides*, *N. gonorrhea*, and secondary syphilis) should be considered. Elevated LFTs should prompt evaluation for infections that might affect the liver such as viral hepatitis, leptospirosis, typhoid fever, and tularemia. Noninfectious causes that cause thrombocytopenia and acute kidney injury should be ruled out as they can be life-threatening and include hemolytic uremic syndrome (HUS), thrombotic thrombocytopenic purpura (TTP), and HLH.

HLH deserves particular attention, not only due to its high mortality but also because it can be caused by AP and can be particularly difficult to differentiate from HGA (90). These two entities share several features such as high levels of interferon (IFN)-gamma, IL-10 and IL-12, and ferritin. In both diseases, the tissue damage results from unregulated cytokine-mediated inflammation, with the severity of the illness correlated with ferritin and IL-12 levels [105].

### 4.5. Treatment and Outcome

Although AP is susceptible to all tetracyclines, the treatment of choice is doxycycline for both pediatric and adult populations [5,8]. The optimal duration of therapy has not been established, although patients usually recover after 7–10 days of treatment [5]. In our systematic review, the mean duration of therapy was 12.9 days, with the longest recorded therapy being 30 days for a patient with AP-related myocarditis and cardiomyopathy [91]. Doxycycline is the drug of choice in the pediatric population as well, with a recommended duration of 5–7 days to avoid dental staining [5].

Rifampin is an alternative treatment for pediatric and pregnant patients. In our review, all patients treated with rifampin demonstrated complete recovery and symptom resolution. Complete recovery was also reported in some patients who received no treatment.

In cases of coinfection with multiple tick-borne pathogens, specific therapy should be prescribed for certain pathogens. Doxycycline is typically effective against *Borrelia* spp., *Rickettsia* spp., and *Ehrlichia* spp., while babesiosis requires the addition of atovaquone and azithromycin or clindamycin and quinine, depending on the severity of illness [8,119,120].

The mortality rate among the cases analyzed in this review was 5.7%, which is significantly higher than the previously reported mortality of 0.6–1% [8,11]. The durations of illness and hospitalization were relatively long, at 20.8 and 14.6 days, respectively. These findings are at least partially related to the fact that severe cases are more likely reported than mild or asymptomatic (publication bias). AP-induced immunosuppression, disease complications, and unusual manifestations encountered in some of the cases also explain the higher mortality and long duration.

The mortality rate in this review was higher in the immunosuppressed group compared to the immunocompetent, 18.2% and 4.2%, respectively. It has been proposed that AP itself may lead to a state of relative immunosuppression resulting in worse clinical outcomes. AP has a strong affinity for infecting and multiplying within WBCs, thereby decreasing their activity and number, and predisposing patients to secondary infections [100,121]. One report described a patient who suffered from chronic lymphocytic leukemia (CLL) [59] and developed myelitis, respiratory distress, nosocomial pneumonia, and shock during their course of HGA. It was uncertain whether myelitis was due to HGA or a paraneoplastic manifestation of CLL. This report testifies how difficult it can sometimes be to delineate symptoms of infection from an underlying disease, especially in patients who are already immunocompromised.

## 5. Limitations of the Study

The limitations of this study are inherent to the nature of the systematic review and the bias that occurs with the selection of the articles. While we implemented strict criteria for the selection of case reports, and did so according to rigorous PRISMA guidelines, we might have inadvertently omitted some high-quality cases that are published in journals not indexed in MEDLINE/PubMed database. Furthermore, because the review consists of case reports and case series only, the mortality and the frequency of unusual disease characteristics, length of stay, and coinfection rates might be skewed as such peculiar cases are potentially more likely to be published.

## 6. Conclusions

HGA is an emerging tick-borne disease with worldwide distribution. While ticks are the main vector, other modes of transmission have been documented such as blood transfusion and transmission by direct contact with infected blood or bodily fluids. Although typically mild (and in some cases indolent), our systematic review demonstrates that HGA can present as a severe illness leading to significant complications, and even death. Presenting signs and symptoms are highly variable, ranging from constitutional symptoms, high fever, gastrointestinal symptoms, rash, and, less commonly, myocarditis, myositis, peripheral neuropathy, and short-term memory impairment, among others. This review demonstrated a particularly high prevalence of GI symptoms (55%) and the prevalence of rash was also higher than previously reported (17%). We found an overall mortality rate of 5.7%, significantly higher than previously described, and that mortality varies between immunocompetent (4.2%) and immunocompromised (18.2%) populations. Additionally, patients may have a long duration of illness and hospitalization. A third of patients who died were not on appropriate antimicrobial therapy, and among those who died who were on appropriate therapy, doxycycline was delayed for more than 48 h from presentation in 50% of patients. Clinicians, especially those treating patients from endemic areas, should maintain a high suspicion for this disease, particularly since appropriate treatment leads to the rapid resolution of symptoms. Further surveillance is needed to monitor how climate change and environmental factors will contribute to the spread of this emerging tick-borne zoonosis.

## Figures and Tables

**Table 1:** Comorbid conditions of immunocompetent patients and cause for immunosuppression in immunocompromised patients.

Comorbid Conditions of Immunocompetent Patients	Number of Patients (%)	Cause for Immunosuppression in Immunocompromised Patients	Number of Patients (%)
Hypertension	9 (8.2)	Steroid use; DM	5 (4.5)
Hypothyroidism; CAD	6 (5.5)	CKD	4 (3.6)
Joint disease	5 (4.5)	Hematologic malignancies; Cytotoxic medication	3 (2.7)
Smoking; HLP; Malignancy	4 (3.6)	Asplenia	1 (0.9)
BPH; COPD; Inherited hematologic abnormality	3 (2.7)		
Hematologic malignancy in remission; HF; Ischemic cardiomyopathy	2 (1.8)		
Other less common comorbidities (hydrocephalus, kidney transplant, A-fib, gynecomastia, depression, Chrons disease, valve replacement, aortic aneurysm, sleep apnea, ureter stones, diverticulosis, colectomy, gunshot wound, SVT, fungal sinusitis, gastritis, hip fracture, fibromyalgia, TIA, embolic stroke, carpal-tunnel syndrome, rhabdomyolysis, Stevens–Johnson syndrome, asthma)	1 (0.9)		

CAD—coronary artery disease, HLP—hyperlipidemia, BPH—benign prostatic hyperplasia, COPD—chronic obstructive pulmonary disease, HF—heart failure, A-fib—atrial fibrillation, SVT—supraventricular tachyarrhythmia, TIA—transitory ischemic attack, DM—diabetes mellitus, CKD—chronic kidney disease.

**Table 2:** Clinical manifestations of the patients diagnosed with HGA.

Symptom		Number of Cases (%)
Fever		99 (90)
Constitutional symptoms		65 (59.1)
GI symptoms	Diarrhea	20 (18.2)
	Nausea	16 (14.5)
	Abdominal pain	10 (9.1)
	Anorexia	8 (7.3)
	Emesis	7 (6.4)
Myalgia		46 (41.8)
Headache		41 (37.3)
Chills		35 (31.8)
Arthralgia		25 (22.7)
Rash		19 (17.3)
AKI		17 (15.5)
Cough		10 (9.1)
Splenic complications	Splenomegaly	10 (9.1)
	Splenic rupture	1 (0.9)
	Splenic infarct	1 (0.9)
Confusion		9 (8.2)
Diaphoresis		8 (7.3)
SOB/Respiratory distress		7 (6.4)
Dizziness		3 (2.7)
Rigor		2 (1.8)

HGA—human granulocytic anaplasmosis, GI—gastrointestinal, AKI—acute kidney injury, SOB—shortness of breath.

**Table 3:** Laboratory investigations and laboratory abnormalities reported in patients diagnosed with HGA.

Parameter	Mean (SD)	Parameter		Number of Patients (%)
Hb ** (g/dL)	12.7 (1.8)	Anemia (<12 g/dL in women, <13 g/dL in men)		33 (30)
Platelets ** (×10 (9)/L)	72 (6.6)	Thrombocytopenia (<150 × 10 (9)/L)		79 (71.8)
WBC ** (×10 (9)/L)	4.8 (0.5)	Leukopenia (<4 × 10 (9)/L)		51 (46.4)
BUN * (mg/dL)	50.1 (8.3)			
Creatinine * (mg/dL)	3.3 (0.4)			
Bilirubin * (mg/dL)	2.3 (0.5)			
AST * (U/L)	162.6 (19.7)	LFT abnormalities	AST (>40 U/L)	63 (57.3)
ALT * (U/L)	107.5 (11.1)		ALT (>50 U/L)	53 (48.2)
ALP * (U/L)	148.4 (23.3)		ALP (>150 U/L)	7 (6.4)
Na ** (mmoL/L)	132.4 (3.1)			
LDH * (U/L)	911.8 (198.5)			
CRP * (mg/dL)	148.1 (14.2)	Inflammation	ESR (>29 mm/h in women, >22 mm/h in men)	13 (11.8)
ESR * (mm/hr)	52.1 (7.4)		CRP (>10 mg/L)	37 (33.6)
Ferritin * (ng/mL)	12,076 (6820)			

* Highest value reported; ** Lowest value reported; HGA—human granulocytic anaplasmosis, Hb—hemoglobin, WBC—white blood cells, BUN—blood urea nitrogen, AST—aspartate aminotransferase, ALT—alanine aminotransferase, ALP—alkaline phosphatase, LDH—lactate dehydrogenase, CRP—C reactive protein, ESR—erythrocyte sedimentation rate, LFT—liver function test.

## Data Availability

Available upon requests from corresponding author.
